# Autoantibodies to a Nodal Isoform of Neurofascin in Pediatric Chronic Inflammatory Demyelinating Polyneuropathy

**DOI:** 10.1177/2329048X221149618

**Published:** 2023-02-15

**Authors:** Eline Chauvet, Geraldine Blanchard Rohner, Véroniqu Manel, Emilien Delmont, Joseph Boucraut, Stephanie Garcia-Tarodo

**Affiliations:** 1 Unit of Neuropediatrics, Department of Women, Children and Adolescent, Geneva University Hospitals, Geneva, Switzerland; 2 Immunology, Department of Women, Children and Adolescent, Geneva University Hospitals, Geneva, Switzerland; 3 Hospices Civils De Lyon, Groupement Hospitalier EST, Hopital Femme Mere Enfant, Bron, France; 4 Referral Centre for Neuromuscular Disease and ALS, La Timone Hospital, Marseille, France; 5 immunology Department, La Conception Hospital, Marseille, France

**Keywords:** pediatric chronic inflammatory demyelinating polyradiculoneuropathy, paranodal antibodies, rituximab

## Abstract

Pediatric chronic inflammatory demyelinating polyradiculoneuropathy (CIDP) is an acquired immune-mediated disorder of the peripheral nervous system with a number of diagnostic pitfalls. A subset of treatment-resistant CIDP adult patients have been found with antibodies against paranodal proteins. We report the first pediatric case in a 14 year-old adolescent with a severe CIDP phenotype in whom positive anti-neurofascin 155 antibodies were found in his serum. Resistant to conventional therapies, he showed dramatic improvement when treated with Rituximab with mild to moderate functional motor disability at 24 month follow-up. In pediatric CIDP patients that remain refractory to conventional treatments, the presence of antibodies to paranodal proteins warrants investigation as it can have potential therapeutic guidance.

## Introduction

Chronic inflammatory demyelinating polyradiculoneuropathy (CIDP) is an acquired immune mediated disorder affecting the peripheral nerves with an insidious course or relapsing episodes. Although less common in children than in adults, revised diagnostic criteria have been outlined for this age group^1^ to facilitate early diagnosis and targeted immunotherapy. Challenges in this population include a larger differential diagnosis, with a number of hereditary and metabolic causes of childhood polyneuropathy, and in most cases, no particular target antigen identified. Recently, in adult patients, antibodies against paranodal proteins are being tested in atypical CIDP phenotypes.^2^ We report the case of a child presenting with CIDP resistant to conventional immunotherapies, who tested positive for anti-neurofascin 155 (NF155) antibodies and subsequently responded to B-cell depletion therapy.

## Case Report

A 14-year-old adolescent, with no previous medical history, presented with fatigue and progressive symmetric lower extremity weakness over a period of 4 weeks. He subsequently lost ambulation and developed upper extremity involvement along with cranial nerve palsies VI, VII, IX, X and XII over the next 2 months. His motor strength was proximally grades 2–3 and distally grades 1–2.Tendon reflexes were absent in all paretic extremities. He also reported distal sensory loss in all extremities. Symptoms culminated in respiratory insufficiency from phrenic involvement and he was transferred to intensive care.

Cerebrospinal fluid (CSF) analysis showed an albumino-cytologic dissociation with a protein level of 2.05g/L and a white blood cell count of 45 cells/mm^3^. An infectious cause was ruled out from negative blood serologies (HIV, Lyme, EBV, E. coli, Campylobacter, CMV, Mycoplasma, Chikungunya, Zika, hepatitis E) and a broad range PCR in the CSF. Serum inflammatory markers found an erythrocyte sedimentation rate of 24mm/h, mildly elevated antinuclear antibodies (1/320) and mildly positive anti-ganglioside IgM antibodies (GM1, GM2 and GD1b). Brain and spinal cord magnetic resonance imagery (MRI) were normal. Electroneuromyography (ENMG) found a severe demyelinating polyradiculoneuropathy with axonal loss. A metabolic and toxic workup was performed, yielding negative results. An ophthalmologic evaluation found bilateral grade III papilledema attributed to secondary intracranial hypertension. An MRI of the lower extremities showed a discrete T2 hyperintensity in the right vastus anterior muscle that was subsequently biopsied to reveal isolated and nonspecific atrophy of type II muscle fibers, with no signs of granulomatous inflammation or abnormal immunohistochemistry markings. A full body PET CT-scan revealed no abnormal hypermetabolism.

He was initially treated with intravenous immunoglobulins at the dose 2g/kg over 5 days, followed by 5 courses of plasmapheresis. Because of continued deterioration, he was given a methylprednisolone bolus of 1g for 3 consecutive days and put on a maintenance dose of oral prednisone. He showed some response to corticosteroids regaining proximal anti-gravitational movements of extremities, improved dysphagia enabling oral feeding, and recuperated comprehensible speech. Methotrexate was added as an initial choice of immunosuppressant therapy given a possible diagnosis of neurosarcoidosis.

Given his progressive course and lack of response to conventional therapies, antibodies to paranodal proteins were investigated in the serum. Anti-NF155 of the IgG4 isotype were detected with a flow cytometry technique using transfected human embryonic kidney (HEK) cells.^2^ He was subsequently started on Rituximab 750mg/m^2^ every two weeks for the first month and then once every 6 months for a period of 18 months. Methotrexate was stopped and he was weaned off steroids. He showed dramatic improvement over the following 12 months. At 24 months follow-up, he is presently able to walk independently with an ankle-foot orthosis bilaterally. He has normal proximal motor strength in the upper and lower extremities. A grade 4 strength is quoted in his distal upper extremities. In his distal lower extremities, he has grade 3 strength for all group muscles, with the exception of his ankle dorsiflexors that remain grade 2. He has regained full sensation and has no remaining cranial nerve deficit. His functional disability amounts to 25/48 on the Rasch-built overall disability scale (R-ODS).

## Discussion

Our patient had a CIDP diagnosis based on his signs of polyneuropathy progressing for more than 8 weeks with a symmetric proximal and distal pattern of weakness. His clinical presentation was severe with sensory deficits and polycranial nerve involvement. Electrophysiological findings were concordant with reduced focal motor and sensory nerve conduction velocities (< 20m/s and < 33m/s respectively) and multiple conduction blocks, along with amplitude reduction in proximal and distal sites. However, response to immunoglobulins and plasmapheresis was dismal, and only partial improvement was seen with high-dose corticosteroids.

The spectrum of adult CIDP has considerably expanded over the past few decades with various clinical subtypes now described.^3^ Recently the discovery of antibodies to paranodal proteins such as contactin-1 (CNTN1) and NF155, have been reported in a subset of CIDP patients with treatment refractoriness to first-line agents. CNTN1 is essential to the organization of the axono-glial junction and NF155 is its glial counterpart. Both proteins play an important role in the structure and function of the node of Ranvier. Antibodies of the IgG4 isotype to these paranodal proteins convey more severe phenotypes^4^ with predominantly distal motor involvement, sensory ataxia, and occasionally a disabling tremor. IgG4 antibodies are produced by regulatory B cells and cannot fix complement or bind to immunoglobulin receptors.^3^ Several reports have suggested a promising response to Rituximab in these cases.^5^

To our knowledge, we report the first pediatric case of CIDP with positive anti-NF155 antibodies, highlighting the importance of including these antibodies in the investigations performed in this age group. In our testing laboratory, this patient was the only pediatric case from a total of 26 patients with positive IgG4 anti-NF 155 antibodies between 2016 and 2022. Furthermore, response to Rituximab was dramatic with mild to moderate residual disability (predominantly distal leg weakness) at 24-month follow-up. He was able to be withdrawn from all other immunosuppressant agents. Another important aspect in our patient's management was that such recovery was obtained despite the introduction of Rituximab at 6 months of disease onset, thus suggesting some effectiveness of this therapy even upon delay in its initiation. A number of points remain to be elucidated in this subtype of CIDP or autoimmune nodopathy as is the current nomenclature, affecting both pediatric and adult populations, including the course of auto-antibody titers throughout disease and recovery and therefore the need for serology monitoring, the duration of Rituximab treatment, and the long-term risk of relapse upon therapy cessation.

In pediatric CIDP patients that remain refractory to conventional treatments, the possible presence of antibodies against paranodal proteins warrants investigation. In case of positive anti-NF155 antibodies of the IgG4 isotype, treatment with Rituximab should be considered.
